# COGNITIVE LOAD IN INDIVIDUALS WITH A TRANSFEMORAL AMPUTATION DURING SINGLE- AND DUAL-TASK WALKING: A PILOT STUDY OF BRAIN ACTIVITY IN PEOPLE USING A SOCKET PROSTHESIS OR A BONE-ANCHORED PROSTHESIS

**DOI:** 10.2340/jrm.v56.40111

**Published:** 2024-08-22

**Authors:** Saffran MÖLLER, Kerstin HAGBERG, Nerrolyn RAMSTRAND

**Affiliations:** 1 Department of Rehabilitation, School of Health and Welfare, Jönköping University, Jönköping, Sweden; 2 Department of Orthopaedics, Sahlgrenska University Hospital, Gothenburg, Sweden; 3 Department of Orthopaedics, Institute of Clinical Sciences, Sahlgrenska Academy, University of Gothenburg, Gothenburg, Sweden

**Keywords:** attention, limb prosthesis, neuroimaging, osseointegration

## Abstract

**Objective:**

To explore cognitive load in people with transfemoral amputations fitted with socket or bone-anchored prostheses by describing activity in the left and right dorsolateral prefrontal cortices during single- and dual-task walking.

**Design:**

Cross-sectional pilot study.

**Patients:**

8 socket prosthesis users and 8 bone-anchored prosthesis users. All were fitted with microprocessor-controlled prosthetic knees.

**Methods:**

Participants answered self-report questionnaires and performed gait tests during 1 single-task walking condition and 2 dual-task walking conditions. While walking, activity in the dorsolateral prefrontal cortex was measured using functional near-infrared spectroscopy. Cognitive load was investigated for each participant by exploring the relative concentration of oxygenated haemoglobin in the left and right dorsolateral prefrontal cortex. Symmetry of brain activity was investigated by calculating a laterality index.

**Results:**

Self-report measures and basic gait variables did not show differences between the groups.

No obvious between-group differences were observed in the relative concentration of oxygenated haemoglobin for any walking condition. There was a tendency towards more right-side brain activity for participants using a socket prosthesis during dual-task conditions.

**Conclusions:**

This pilot study did not identify substantial differences in cognitive load or lateralization between socket prosthesis users and bone-anchored prosthesis users.

## LAY ABSTRACT

For able-bodied individuals, the act of walking requires little cognitive effort; for people who need a prosthesis to walk, more cognitive effort is required. Prosthesis users are also less proficient at performing secondary tasks while they walk. This study assessed the cognitive effort required to walk in 2 groups of people with amputations above the knee. One group were fitted with prostheses incorporating a rigid socket (8 participants) while the other group were fitted with prostheses that were directly attached to the residual skeleton (8 participants). Cognitive effort was measured using a neuroimaging system recording activity in the frontal cortex of the brain. Increased activity in this area of the brain is associated with increased cognitive effort. Results showed no substantial differences in brain activity between the 2 groups of prosthesis users. This information is of importance when advising patients who are considering undergoing treatment for a bone-anchored prosthesis.

Under favourable conditions, maintenance of balance and walking are largely controlled by postural responses at sub-cortical levels and require minimal attentional resources (1). If balance and posture are compromised, as is the case for lower-limb prosthesis users, it becomes necessary to allocate more attention at the perceptual level, requiring activation of cortical areas in the brain. The dorsolateral pre-frontal cortex (dlPFC), responsible for working memory and executive functions, is one such area (2). The explicit postdictive engagement of working memory and executive functions generates *cognitive load* and is perceived as *effort* (3). Excessive cognitive load can have negative effects on task performance and is associated with cognitive fatigue (4).

Traditionally, lower-limb prostheses are designed with a rigid socket encompassing the residual limb (socket prosthesis). In these prostheses, forces are transferred to and from the prosthesis via the soft tissues surrounding the residual skeleton. Any movement occurring between the socket and the residuum (e.g., distal translation or pistoning) creates inefficiency in the system and is considered undesirable (5).

Bone-anchored prostheses (BAPs) are an alternative to socket prostheses and are attached directly to an intramedullary implant osseointegrated in the residual skeleton. Direct skeletal attachment is advantageous for people experiencing problems maintaining suspension of their device or having issues with skin breakdown or discomfort due to a socket (6).

Studies comparing BAP's to socket prosthesis users have reported similar results for walking speed and cadence (7, 8) but conflicting results when it comes to measurement of balance. Gailey et al. (7) reported no difference in self-reported balance confidence, measured using the Activities-specific Balance Confidence Scale (ABC) while Gaffney et al. (9), using the same outcome measure, reported more positive results for BAP users, suggesting the difference was likely due to improved proprioception with a BAP. An improved ability to detect vibrations among BAP users has also been recorded (10).

When balance is challenged in able-bodied individuals, by altering surface stability or adding a secondary task (dual-task), neural activity in the pre-frontal cortex (PFC) increases. A systematic review of studies measuring brain activity during walking (11) in older adults concluded that activity in the PFC is higher during dual-task walking (12). Research comparing socket prosthesis users with a group of able-bodied individuals provides evidence to suggest that walking with a prosthesis is also a cognitively challenging task, with prosthesis users activating the PFC to a greater extent than their able-bodied peers (11). Interestingly, the type of prosthetic knee unit used by prosthesis users has been found to affect PFC activity, with users of microprocessor-controlled prosthetic knees recording lower levels of activity during walking when compared with users of mechanical knee joints (11).

Challenging walking conditions have also been linked to lateralization in the PFC. St George et al. (13) demonstrated that left side dominant activity in the PFC becomes increasingly bilateral when walking becomes more challenging. This is believed to result from a need for additional cognitive processing and suggests that the PFC has a compensatory role in maintaining postural stability.

The aim of this study was to explore cognitive load in people with a transfemoral amputation fitted with a socket or a bone-anchored prosthesis by describing activity and lateralization in the dorsolateral prefrontal cortex during single- and dual-task walking.

## METHOD

This was an observational pilot study involving existing prosthesis users and using a cross-sectional design describing 2 groups. Participants were not prospectively assigned to specific interventions and as such the research was not registered as a clinical trial. To compare characteristics of the 2 groups, gait variables and self-report measures of mobility and balance confidence were collected. Activity in the dlPFC was measured using functional near-infrared spectroscopy (fNIRS) under 3 conditions: 1 single-task walking condition and 2 different dual-task walking conditions. fNIRS estimates of cortical brain activity were derived by measuring the relative concentration of oxygenated haemoglobin (HbO_2_) in the brain (14). Activity in both the left and right dlPFC of the brain were recorded.

### Participants

Participants with a unilateral TFA using a BAP were recruited via the university hospital where their surgical treatment for a BAP had been performed. All had had an OPRA implant (Integrum AB, Mölndal, Sweden) for a minimum of 2 years and lived in Sweden. Data for participants with a unilateral TFA using a socket prosthesis were taken from a previous study (11). For inclusion, participants were required to be able to walk 500 m with the support of no more than a single cane or crutch, to currently use a microprocessor-controlled prosthetic knee joint, and to not have any other impairment or disease that could affect their gait. Individuals with cognitive impairment (Mini-Mental State Examination < 27 (15) were excluded. Prosthetic knees were standardized to include only microprocessor-controlled joints as these are commonly prescribed to users of BAPs. The study was approved by the Regional Ethics Committee in Linköping, Sweden (Dnr 2015/1526, Dnr 2018/289-32). Written informed consent was received from all participants.

### Procedure

Participants were asked to take part in 1 single testing occasion lasting for approximately 2 h. Testing took place at a rehabilitation centre or in a gait laboratory depending on the most convenient location for each participant. Testing locations were quiet and free from activities that might otherwise distract the participant. Testing sessions were initiated by collecting participants’ demographic data and requesting them to complete questionnaires on self-reported prosthetic mobility and balance confidence as well as performing tests measuring ambulation and functionality (described below).

### Self-report questionnaires

Three validated self-report questionnaires were used to describe the prosthetic mobility: (*i*) the Q-TFA Prosthetic Use Score (16), (*ii*) the Activities-Specific Balance Confidence Scale (ABC) (17), and (*iii*) the Prosthetic Limb Users Survey of Mobility (PLUS-M^TM^) 12-item Short Form (18). The Prosthetic Use score (scored 0–100) measured the amount of time the prosthesis users wore their device during a normal week. The ABC (scored 0–100) was used to evaluate perceived balance confidence in performing daily activities and the PLUS-M (expressed as a T-score between 21.8 and 71.4) was used to assess the prosthesis users’ perceived ability to ambulate using a prosthesis in daily activities.

### Gait tasks

fNIRS data were collected while participants walked back and forth along a 14-m level walkway that was free from distractions. Participants were instructed to walk at a self-selected speed and were permitted to use one mobility aid if they wished, i.e., a crutch or stick. The single-task walking condition involved walking on a hinder-free level track, while the dual-task walking conditions included walking while finding numbered and coloured keys (key-test) (19) and a modified trail-walking test (TWT) (20). These specific dual-task activities were selected as they have been used previously in studies with lower-limb prosthesis users. All 3 conditions have been described in detail in a previous publication (21). Each condition was repeated 4 times.

Time and number of steps over the first 10 m of single-task walking were recorded to determine cadence. Upon completion of all walking conditions the fNIRS system was removed and the participant performed a 6-Minute Walk Test (6MWT). This test has been identified as a valid measure of ambulation and functional level in lower-limb prosthesis users (22). The 6MWT was performed once on a 25-m stretch of walkway and the total distance travelled was measured to the closest metre.

### fNIRS data acquisition

**Fig. 1:**
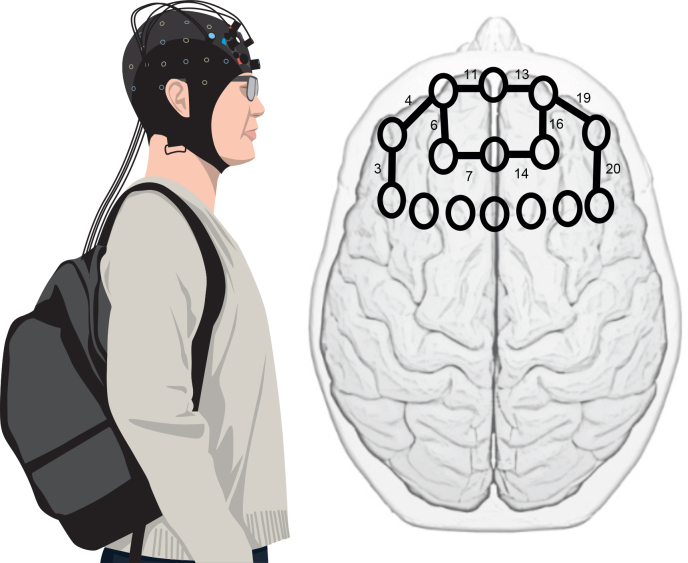
Illustration of the fNIRS equipment and placement of optodes. Left: equipment worn by the participants. A backpack including a laptop and an elasticized cap to secure the optodes. Right: circles define placement of the 16 optodes (8 sources and 8 detectors) covering both hemispheres of the pre-frontal cortex. Black lines define the channels selected (left 3, 4, 6, 7, 11 and right 13, 14, 16, 19, 20) to represent the region of the dorsolateral prefrontal cortex.

To capture cortical brain activity during walking a portable, continuous wave, NIRSSport tandem fNIRS system was used, (NIRx Medical Technologies LLC, NY, USA). This required participants to be fitted with an elastic cap in which 16 optodes were positioned to cover both hemispheres of the PFC (Fig. 1, right). Optodes were positioned according to the International 10–20 System (23). To maximise reliability of Optode placement, measurements and positioning were always performed by the same investigator (SM). The coordinate system used to measure for cap size and to position optodes followed recommendations from Oostenvald and Praamstra (24). Optodes were tethered to a laptop computer placed in a backpack worn by the participant (Fig. 1, left). Data were captured using NIRStar acquisition software (NIRx Medical; NIRx Medical Technologies LLC, Glen Head, NY, USA).

The fNIRS testing procedure began with a 30 s baseline measurement, performed in accordance with the recommendations of Herold et al. (25) and during which the participants sat in silence with their eyes closed. After the baseline measurement participants were asked to stand for a further 30 s to allow the signals to return to pre-test levels and avoid non-linear effects of the haemodynamic refractory period.

### fNIRS data processing

NirsLAB 2017.06 software (NIRx Medical Technologies LLC, NY, USA) was used for post processing of fNIRS data. The first walking trial of each condition was considered a practice trial and removed from the data set while 10 s of data were extracted from each of the remaining 3 trials. The 10 s period did not include the first 5 s of walking as this period would have included a period of acceleration, which may have placed a greater demand on the PFC. In this pilot study we chose to extract data only for oxygenated haemoglobin (HbO_2_) as this is where the largest variations in signal amplitude are likely to be seen (26).

Processing of fNIRS data began by calculating the coefficient of variation (CV) for the unfiltered channels and estimating the signal-to-noise performance. Possible sources of an increased CV are relative motion between the optodes and the tissue of the scalp, as well as physiological artifacts such as blood-pressure induced haemodynamics (27). Potential artefacts were addressed by removing channels with a CV greater than 7.5%.

A bandpass filter (0.01 to 0.2Hz) was then used to eliminate fluctuations related to external factors such as respiration, heartbeat, and low-frequency signal drift. Signals were converted from light intensity levels to concentrations of haemoglobin using the modified Beer–Lambert Law (28) and then normalized to baseline values.

Channels representing the left dlPFC (channels 3, 4, 6, 7, 11) and the right dlPFC (channels 13, 14, 16, 19, 20) were averaged to create 2 regions of interest (ROI) (Fig. 1, right). These regions were then averaged across the 3 x 10 s sets of data that had been extracted for each of the 3 test conditions. To assess the degree of lateralization we calculated a laterality index to reflect symmetry of cortical brain activity in the ROI of the left and right sides. Several different formulae to calculate the laterality index have been proposed in the literature. As use of 1 formula over another does not appear to alter outcomes (29), we chose to use the index published by St George et al. (13), calculated using the formula below. Positive values indicate greater activity in the left cortical region relative to the right, whereas negative values indicate greater activity in the right cortical region relative to the left.


$$Laterality\;index=\frac{\text{left D }\left[{\text{HbO}}_{2}\right]-\text{right D }\left[{\text{HbO}}_{2}\right]}{\text{left D }\left[{\text{HbO}}_{2}\right]+\text{right D }\left[{\text{HbO}}_{2}\right]}$$

### Statistical analysis

IBM SPSS Statistics 21 (IBM Corp, Armonk, NY, USA) was used for all analyses. Demographic and descriptive data are presented as means and standard deviations as well as median and minimum/maximum values. To assess potential differences between the 2 groups (socket prosthesis and BAP users) in regard to demographic and descriptive data a Man–Whitney U test was used. The critical alpha level was set at *p* < 0.05.

In this pilot study fNIRS-average data are presented as graphs illustrating left and right dlPFC activity (HbO_2_) for each participant (socket prosthesis users and BAP users) and each condition (single-task walking, key-test and TWT).

## RESULTS

Participant demographics including age, sex, time since amputation, cause of amputation, height, and current prosthetic knee component are summarized in Table I. Eight participants used a socket prosthesis (1 woman, 7 men, mean age 46 years) and 8 used a BAP (1 woman, 7 men, mean age 52 years). The main cause of amputation among participants was trauma or tumour and the mean time since amputation was 23 years in the group using a socket prosthesis and 16 years in the BAP group. The participants using a BAP had received osseointegration treatment between 4 and 16 years prior to testing. No statistically significant differences were found between the 2 groups in relation to demographic data (Table I), self-reported data (prosthetic use score, ABC, and PLUS-M) or gait data (cadence and 6MWT) (Table II).

**Table I:** Participant details

Groups	Age (years)	Sex	Time since amputation (years)	Cause of amputation	Height (cm)	Prosthetic knee component
Socket prosthesis	56	Male	39	Tumour	180	Genium^a^
	51	Male	33	Tumour	180	Rheo Knee^®b^
	54	Male	46	Tumour	178	Rheo Knee^®b^
	54	Male	43	Tumour	183	C-leg^a^
	32	Male	6	Trauma	180	Genium^a^
	38	Female	3	Tumour	178	Rheo Knee^®b^
	46	Male	9	Tumour	181	C-leg^a^
	35	Male	8	Trauma	180	Rheo Knee^®b^
*Mean (SD)*	*46 (9.5)*		*23 (18.5)*		*180 (1.4)*	
BAP	63	Male	10	Trauma	188	C-leg^a^
	62	Male	6	Trauma	190	Genium^a^
	52	Male	13	Trauma	187	Genium^a^
	27	Male	10	Trauma	187	C-leg^a^
	47	Male	16	Trauma	188	Genium^a^
	77	Male	42	Tumour	178	C-leg^a^
	41	Male	19	Tumour	187	C-leg^a^
	48	Female	13	Trauma	167	Genium^a^
*Mean (SD)*	*52 (15.3)*		*16 (11.2)*		*184 (7.7)*	
*p*-value	0.442		0.009		0.083	

a Ottobock,

b Ossur.

BAP: bone-anchored prosthesis; SD: standard deviation.

**Table II:** Description of self-reported prosthetic mobility, balance confidence, and basic gait tasks and comparisons between the groups

Outcome measures	Socket prosthesis Mean (SD) Median (min–max)	BAP Mean (SD) Median (min–max)	*p*-value
Prosthetic use score	94 (11.0) 100 (71–100)	93 (13.4) 100 (71–100)	0.890
ABC	88 (9.2) 93 (74–98)	79 (8.78) 81 (63–89)	0.130
PLUS-M	56.8 (6.5) 56.3 (48.4–67.1)	51.8 (5.2) 51.6 (44.5–61.0)	0.161
Cadence	102 (8.3) 100 (92–112)	99 (9.67) 99 (80–111)	0.505
6MWT	548 (166) 470 (412–806)	412 (59.7) 420 (310–497)	0.094

SD: standard deviation. BAP: bone-anchored prosthesis. 6MWT: 6-Minutes’ Walk Test. PLUS-M: Prosthetic Limb Users Survey of Mobility. ABC: Activity Balance Confidence Scale.

**Fig. 2:**
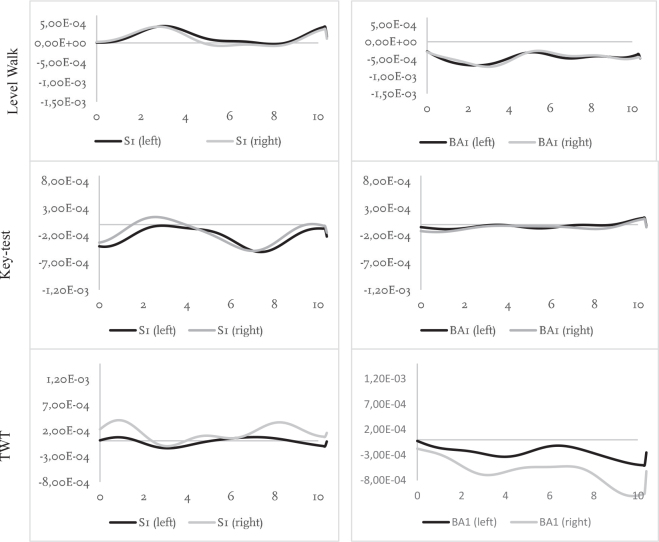
Illustrating cortical brain activity (millimoles of oxygenated haemoglobin, HbO_2_) in the left and right regions of interest in 1 individual from each group and for each walking condition. The Y axis represents the relative concentration of HbO_2_ (mM). The X axis represents time (s). S: socket prosthesis user. BA: bone-anchored prosthesis user. TWT: trail-walking test.

Fig. 2 illustrates an example of fNIRS data for 1 participant from each group and presents mean activity in the left and right dlPFC (HbO_2_) for each walking condition. Graphs for all participants are seen in Figs S1–S3; due to technical difficulties data for 2 participants were missing. During the single-task walking condition, no obvious differences could be seen between socket prosthesis users and BAP users, with the amplitude of haemodynamic signals (HbO_2_) being similar for each group. Haemodynamic signals during the 2 dual-task conditions, the key-test and the TWT, were also relatively similar between the 2 groups. In these dual-task conditions both groups showed higher level fluctuations in amplitude of the haemodynamic signal as compared with single-task walking.

**Fig. 3:**
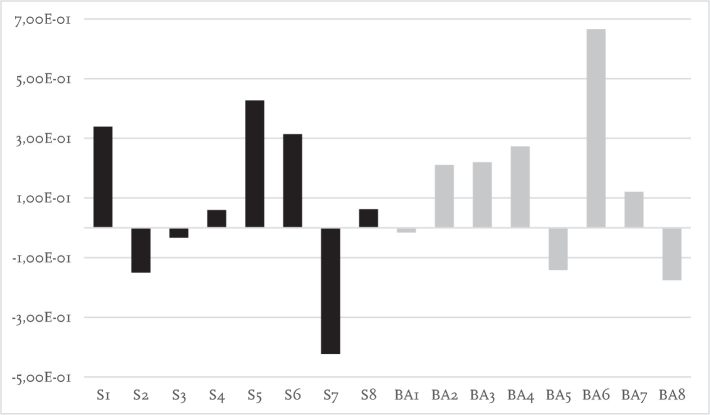
Laterality index for level walking showing activation symmetry between left and right cortical regions (millimoles of oxygenated haemoglobin, HbO_2_). Positive values indicate greater activity in the left cortical region relative to the right, whereas negative values indicate greater activity in the right cortical region relative to the left. The Y axis represents the relative concentration of HbO_2_ (mM). The X axis represents participants. S: socket prosthesis user. BA: bone-anchored prosthesis user.

**Fig. 4:**
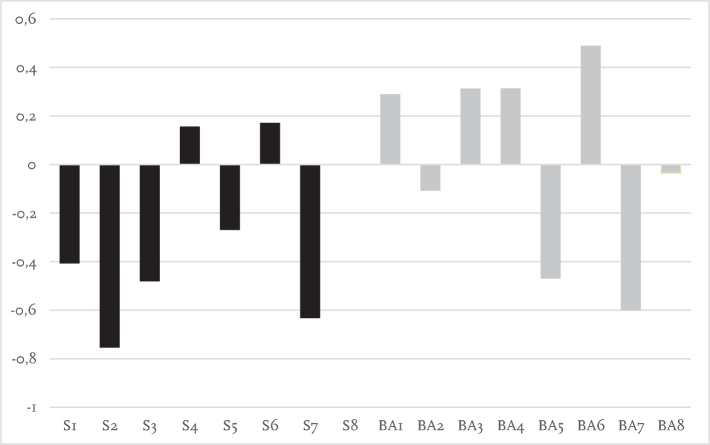
Laterality index for key-test showing activation symmetry between left and right cortical regions (millimoles of oxygenated haemoglobin, HbO_2_). Positive values indicate greater activity in the left cortical region relative to the right, whereas negative values indicate greater activity in the right cortical region relative to the left. The Y axis represents the relative concentration of HbO_2_ (mM). The X axis represents participants. S: socket prosthesis user. BA: bone-anchored prosthesis user. Note S8 missing data.

**Fig. 5:**
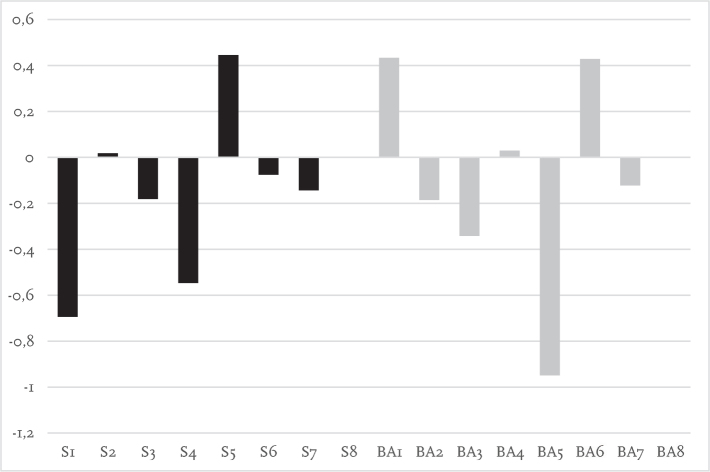
Laterality index for trail-walking test (TWT) showing activation symmetry between left and right cortical regions (millimoles of oxygenated haemoglobin, HbO_2_). Positive values indicate greater activity in the left cortical region relative to the right, whereas negative values indicate greater activity in the right cortical region relative to the left. The Y axis represents the relative concentration of HbO_2_ (mM). The X axis represents participants. S: socket prosthesis user. BA: bone-anchored prosthesis user. Note S8 and BA8 missing data.

Laterality indices for each participant are presented in Figs 3–5. During single-task walking most participants had left lateralized dlPFC activity with no obvious differences between participants using socket prosthesis and BAPs. For both dual-task walking conditions (key-test and TWT) there appeared to be more right side dlPFC activity when compared with the single-task condition. For the key-test condition there were more socket prosthesis users with right lateralized dlPFC as compared with BAP users. This was not the case in the TWT condition.

## DISCUSSION

To the authors’ knowledge, this is the first study to describe cognitive load by measuring HbO_2_ during walking in people with a unilateral transfemoral amputation using a BAP.

Except for the coupling between the prosthesis and the residuum the 2 groups included in this study were considered comparable. All were established prosthesis users, fitted with the same category of prosthetic knee mechanism, and used their prosthetic limb to a high degree. No significant group differences were observed in measures related to prosthetic mobility.

With group equivalence established, the primary aim of our pilot study was to explore cognitive load in socket prosthesis users versus BAP users by measuring cortical brain activity in the left and right dlPFC. The association between increased PFC activity and an increase in cognitive demand is well established (30) and higher amplitude HbO_2_ signals reported when comparing prosthesis users with able-bodied controls suggest that the cognitive resources required for walking are greater in this population (11). In the present study, haemodynamic activity in the dlPFC, during both single- and dual-task walking conditions, was similar between the two groups (i.e., no group displayed signals that were consistently higher or lower than the other). While these fNIRS data suggest little difference in cognitive load between the groups it should be recognized that fNIRS technology measures cortical activity in the order of seconds and does not allow for discrimination of events that may occur within a gait cycle (31). Analysis of events within a gait cycle is better addressed using electroencephalography (EEG), which has lower spatial resolution when compared with fNIRS but can measure electrophysiological brain activation within milliseconds (31). Preliminary EEG data comparing BAP and socket prosthesis users have provided some evidence to suggest that there may be differences in brain activity that occur around the time of heel strike, but further research is required to confirm this preliminary finding (32).

The left dlPFC has been linked to planning of motor sequences and allocation of attention (33). The goal of calculating a laterality index in the present study was subsequently to determine whether there was left or right-side hemispheric dominance that may distinguish between socket prosthesis and BAP users. In previous work, St George et al. (13) indicated that haemodynamic activity is more lateralized during easy balance tasks but, due to the need to engage more cognitive resources during challenging tasks, activity becomes more bilateral during more difficult tasks. During single-task walking, both the socket prosthesis and BAP groups in this study displayed largely left lateralized dlPFC activity. This suggests that neither group found it necessary to engage additional cognitive resources to complete the single-task walking condition. In addition, no obvious group differences were observed during the dual-task conditions; however, it is worth noting that both groups included more participants with right side dlPFC laterality. This finding is consistent with dual-task studies conducted on other populations (34). Right side laterality has been suggested to result from increased activity in the ventral attentional network, which aligns with the right PFC (34). The ventral attentional network is responsible for swapping attention between tasks (35) and would likely have been active during both the key-test and the TWT in the present study.

While this pilot study did not identify any major differences in cortical brain activity between socket prosthesis users and BAP users it is important to recognize that differences have previously been identified in other types of outcome measures. Qualitative results suggest that users of BAPs experience an increased feeling of their prosthesis being “an incorporated part of their body” (36). Quantitative studies have shown improvement in osseoperception, measured through the user’s ability to detect vibrations applied to the prosthesis (10), as well as more normal neuromuscular function in BAP users (37). Previous research has also shown that people who had experienced bothersome problems with a socket prosthesis and have transitioned to a BAP use their prosthesis more, and have improved mobility and an enhanced quality of life (38, 39). Given the substantial changes reported in individuals transitioning from a socket prosthesis to a BAP and challenges to ensure homogeneity between groups of socket prostheses users and BAP users, we recommend that future studies investigate cognitive load prospectively within subjects.

### Limitations

Pilot studies, such as the present study, represent a fundamental phase of the research process by allowing researchers to examine the feasibility of recruitment procedures, assessment procedures, and methods for potential larger scale studies. As a pilot study, results of the present research are limited by a small sample size, which does not allow for testing of hypotheses or generalization of results (40). A further limitation of this study is our failure to control for the residual limb length of participants. Residual limb length is known to have a substantial influence on the gait outcomes of transfemoral prosthesis users (41) and a short residual limb is often an underlying reason why prosthesis users choose to undergo an osseointegration procedure (6). As a result, it is likely that participants in this study who were fitted with a socket prosthesis had longer residual limbs than those fitted with a BAP. The type of knee joint used in a prosthesis has previously been demonstrated to affect PFC activity (11). To control the potential effects of knee joint prescription we chose to standardize participants’ knees to include only microprocessor-controlled joints. We did not, however, control for the specific type of microprocessor-controlled joint used by each individual and it is possible that this may account for some variance in PFC activity between participants.

The dual-task activities assessed in this pilot study were selected on the basis that they had been used in previous publications involving prosthesis users. The type of dual task performed during gait is, however, known to have an effect on brain activity (42, 43) and it is likely that selection of other tasks may have elicited different results.

While fNIRS offers a portable, non-invasive means of monitoring brain activity it does have some limitations, which may contribute to bias. These are largely related to artefacts caused by motion or physiological noise (44, 45). For example, blood flow changes in the extracerebral layers of the head are known to interfere with fNIRS signals. This can be addressed by using short-separation reference channels; however, we did not have access to short separation channels at the time these data were collected. As this is a pilot study, which will be used to determine whether future, large scale, studies are warranted, we limited our analysis to measures of oxygenated haemoglobin as this is where one would expect to see the largest variations in signal amplitude. To gain a complete picture of cortical brain activity, we recommend that any future studies include data related to total haemoglobin concentrations and deoxygenated haemoglobin concentrations.

### Conclusion

The findings in this pilot study did not identify substantial differences in cognitive load and lateralization between socket prosthesis and BAP users, as measured using fNIRS during single- and dual-task walking conditions. Larger scale studies will be needed to confirm results, while controlling for residual limb length as an important variable that affects gait outcomes. We also recommend that future research, with larger sample sizes, continues to investigate activation symmetry between the left and right sides of the brain in this population as well as other groups with inherent balance and stability problems.

## Supplementary Material

COGNITIVE LOAD IN INDIVIDUALS WITH A TRANSFEMORAL AMPUTATION DURING SINGLE- AND DUAL-TASK WALKING: A PILOT STUDY OF BRAIN ACTIVITY IN PEOPLE USING A SOCKET PROSTHESIS OR A BONE-ANCHORED PROSTHESIS

## References

[1] Horak FB. Postural control. In: Binder MD, Hirokawa N, Windhorst U, editors. Encyclopedia of Neuroscience. Berlin, Heidelberg: Springer Berlin Heidelberg; 2009: p. 3212–3219. DOI: 10.1007/978-3-540-29678-2_4708

[2] Sturm V, Haase CM, Levenson RW. Emotional dysfunction in psychopathology and neuropathology: neural and genetic pathways. In: Lehner T, Miller BL, State MW, editors. Genomics, circuits, and pathways in clinical neuropsychiatry. Cambridge, MA: Elsevier Academic Press. p. 345–364. DOI: 10.1016/B978-0-12-800105-9.00022-6

[3] Rudner M, Lunner T, Behrens T, Thorén ES, Rönnberg J. Working memory capacity may influence perceived effort during aided speech recognition in noise. J Am Acad Audiol 2012; 23: 577–589. DOI: 10.3766/jaaa.23.7.7 22967733

[4] Aben B, Buc Calderon C, Van den Bussche E, Verguts T. Cognitive effort modulates connectivity between dorsal anterior cingulate cortex and task-relevant cortical areas. J Neurosci 2020; 40: 3838–3848. DOI: 10.1523/jneurosci.2948-19.2020 32273486 PMC7204076

[5] Maikos JT, Chomack JM, Loan JP, Bradley KM, D’Andrea SE. Effects of prosthetic socket design on residual femur motion using dynamic stereo X-ray: a preliminary analysis. Front Bioeng Biotechnol 2021; 9: 697651. DOI: 10.3389/fbioe.2021.697651 34447740 PMC8383143

[6] Li Y, Felländer-Tsai L. The bone anchored prostheses for amputees: historical development, current status, and future aspects. Biomaterials 2021; 273: 120836. DOI: 10.1016/j.biomaterials.2021.120836 33894405

[7] Gailey RS, Kristal A, Al Muderis M, Lučarević J, Clemens S, Applegate EB, et al. Comparison of prosthetic mobility and balance in transfemoral amputees with bone-anchored prosthesis vs. socket prosthesis. Prosthet Orthot Int 2023; 47: 130–136. DOI: 10.1097/pxr.0000000000000189 36701197

[8] Ranaldi S, Naaim A, Marchis C, Robert T, Dumas R, Conforto S, et al. Walking ability of individuals fitted with transfemoral bone-anchored prostheses: a comparative study of gait parameters. Clin Rehabil 2023; 37: 1670–1683. DOI: 10.1177/02692155231183779 37350084 PMC10580681

[9] Gaffney BMM, Davis-Wilson HC, Christiansen CL, Awad ME, Lev G, Tracy J, et al. Osseointegrated prostheses improve balance and balance confidence in individuals with unilateral transfemoral limb loss. Gait Posture 2023; 100: 132–138. DOI: 10.1016/j.gaitpost.2022.12.011 36521257

[10] Orgel M, Elareibi M, Graulich T, Krettek C, Neunaber C, Aschoff HH, et al. Osseoperception in transcutaneous osseointegrated prosthetic systems (TOPS) after transfemoral amputation: a prospective study. Arch Orthop Trauma Surg 2023; 143: 603–610. DOI: 10.1007/s00402-021-04099-1 34345935

[11] Moller S, Rusaw D, Hagberg K, Ramstrand N. Reduced cortical brain activity with the use of microprocessor-controlled prosthetic knees during walking. Prosthet Orthot Int 2019; 43: 257–265. DOI: 10.1177/0309364618805260 30375285

[12] Kahya M, Moon S, Ranchet M, Vukas RR, Lyons KE, Pahwa R, et al. Brain activity during dual task gait and balance in aging and age-related neurodegenerative conditions: a systematic review. Exp Gerontol 2019; 128: 110756. DOI: 10.1016/j.exger.2019.110756 31648005 PMC6876748

[13] St George RJ, Hinder MR, Puri R, Walker E, Callisaya ML. Functional near-infrared spectroscopy reveals the compensatory potential of pre-frontal cortical activity for standing balance in young and older adults. Neuroscience 2021; 452: 208–218. DOI: 10.1016/j.neuroscience.2020.10.027 33197501

[14] Pelicioni PHS, Tijsma M, Lord SR, Menant J. Prefrontal cortical activation measured by fNIRS during walking: effects of age, disease and secondary task. PeerJ 2019; 7: e6833. DOI: 10.7717/peerj.6833 31110922 PMC6501770

[15] Palmqvist S, Hansson O, Minthon L, Londos E. Practical suggestions on how to differentiate dementia with Lewy bodies from Alzheimer’s disease with common cognitive tests. Int J Geriatr Psychiatry 2009; 24: 1405–1412. DOI: 10.1002/gps.2277 19347836

[16] Ting A, Edwards LB. Human leukocyte antigen in the allocation of kidneys from cadaveric donors in the United States. Transplantation 2004; 77: 610–614. DOI: 10.1097/01.tp.0000103724.27166.ad 15084946

[17] Powell LE, Myers AM. The Activities-specific balance confidence (ABC) scale. J Gerontol A Biol Sci Med Sci 1995; 50a: M28–34. 7814786 10.1093/gerona/50a.1.m28

[18] Hafner BJ, Morgan SJ, Askew RL, Salem R. Psychometric evaluation of self-report outcome measures for prosthetic applications. J Rehabil Res Dev 2016; 53: 797–812. DOI: 10.1682/jrrd.2015.12.0228 28273329 PMC5345485

[19] Howard CL, Wallace CT, Rock M, Stokic DS. Dual task gait analysis in prosthesis users. Paper presented at the 39th Academy Annual Meeting and Scientific Symposium, American Academy of Orthotists & Prosthetists 2013.

[20] Yamada M, Ichihashi N. Predicting the probability of falls in community-dwelling elderly individuals using the trail-walking test. Environ Health Prev Med 2010; 15: 386–391. DOI: 10.1007/s12199-010-0154-1 21432571 PMC2955901

[21] Möller S, Ramstrand N, Hagberg K, Rusaw D. Cortical brain activity in transfemoral or knee-disarticulation prosthesis users performing single- and dual-task walking activities. J Rehabil Assist Technol Eng 2020; 7: 2055668320964109. DOI: 10.1177/2055668320964109 33224519 PMC7649851

[22] Reid L, Thomson P, Besemann M, Dudek N. Going places: does the two-minute walk test predict the six-minute walk test in lower extremity amputees? J Rehabil Med 2015; 47: 256–261. DOI: 10.2340/16501977-1916 25588644

[23] Okamoto M, Dan H, Sakamoto K, Takeo K, Shimizu K, Kohno S, et al. Three-dimensional probabilistic anatomical cranio-cerebral correlation via the international 10–20 system oriented for transcranial functional brain mapping. Neuroimage 2004; 21: 99–111. DOI: 10.1016/j.neuroimage.2003.08.026 14741647

[24] Oostenveld R, Praamstra P. The five percent electrode system for high-resolution EEG and ERP measurements. Clin Neurophysiol 2001; 112: 713–719. DOI: 10.1016/s1388-2457(00)00527-7 11275545

[25] Herold F, Wiegel P, Scholkmann F, Müller NG. Applications of functional near-infrared spectroscopy (fNIRS) neuroimaging in exercise cognition science: a systematic, methodology-focused review. J Clin Med 2018; 7. DOI: 10.3390/jcm7120466 PMC6306799 30469482

[26] Hoshi Y. Hemodynamic signals in fNIRS. Prog Brain Res 2016; 225: 153–179. DOI: 10.1016/bs.pbr.2016.03.004 27130415

[27] Piper SK, Krueger A, Koch SP, Mehnert J, Habermehl C, Steinbrink J, et al. A wearable multi-channel fNIRS system for brain imaging in freely moving subjects. Neuroimage 2014; 85 Pt 1: 64–71. DOI: 10.1016/j.neuroimage.2013.06.062 23810973 PMC3859838

[28] Baker WB, Parthasarathy AB, Busch DR, Mesquita RC, Greenberg JH, Yodh AG. Modified Beer-Lambert law for blood flow. Biomed Opt Express 2014; 5: 4053–4075. DOI: 10.1364/BOE.5.004053 25426330 PMC4242038

[29] Borrell JA, Fraser K, Manattu AK, Zuniga JM. Laterality index calculations in a control study of functional near infrared spectroscopy. Brain Topogr 2023; 36: 210–222. DOI: 10.1007/s10548-023-00942-3 36757503

[30] Wang Q, Dai W, Xu S, Zhu S, Sui Y, Kan C, et al. Brain activation of the PFC during dual-task walking in stroke patients: a systematic review and meta-analysis of functional near-infrared spectroscopy studies. Front Neurosci 2023; 17: 1111274. DOI: 10.3389/fnins.2023.1111274 36875661 PMC9980909

[31] Tong L. Evaluation of different brain imaging technologies. In: Khalil R. Viti C, Cui MY, Hakobyan H, editors. Proceedings of the 2021 International Conference on Public Art and Human Development (ICPAHD 2021), Advances in Social Science, Education and Humanities Research series. London: Atlantis Press/Springer Nature; 2021. p. 692–696. DOI: 10.2991/assehr.k.220110.132

[32] Kooiman V, van der Cruijsen J, Leijendekkers R, Verdonschot N, Solis-Escalante T, Weerdesteyn V. The influence of prosthetic suspension on gait and cortical modulations is persons with a transfemoral amputation: socket-suspended versus bone-anchored prosthesis. J Neuroeng Rehabil 2024; 21: 35. DOI: 10.1186/s12984-024-01331-y 38454427 PMC10921721

[33] Kaller CP, Rahm B, Spreer J, Weiller C, Unterrainer JM. Dissociable contributions of left and right dorsolateral prefrontal cortex in planning. Cereb Cortex 2011; 21: 307–317. DOI: 10.1093/cercor/bhq096 20522540

[34] Rahman TT, Polskaia N, St-Amant G, Salzman T, Vallejo DT, Lajoie Y, et al. An fNIRS investigation of discrete and continuous cognitive demands during dual-task walking in young adults. Front Hum Neurosci 2021; 15: 711054. DOI: 10.3389/fnhum.2021.711054 34867235 PMC8637836

[35] Alves PN, Forkel SJ, Corbetta M, Thiebaut de Schotten M. The subcortical and neurochemical organization of the ventral and dorsal attention networks. Commun Biol 2022; 5: 1343. DOI: 10.1038/s42003-022-04281-0 36477440 PMC9729227

[36] Lundberg M, Hagberg K, Bullington J. My prosthesis as a part of me: a qualitative analysis of living with an osseointegrated prosthetic limb. Prosthet Orthot Int 2011; 35: 207–214. DOI: 10.1177/0309364611409795 21697203

[37] Krauskopf T, Lauck T, Meyer B, Klein L, Mueller M, Kubosch J, et al. Neuromuscular adaptations after osseointegration of a bone-anchored prosthesis in a unilateral transfemoral amputee: a case study. Ann Med 2023; 55: 2255206. DOI: 10.1080/07853890.2023.2255206 37677026 PMC10486294

[38] Hebert JS, Rehani M, Stiegelmar R. Osseointegration for lower-limb amputation: a systematic review of clinical outcomes. JBJS Rev 2017; 5: e10. DOI: 10.2106/JBJS.RVW.17.00037 29087966

[39] Hagberg K, Hansson E, Brånemark R. Outcome of percutaneous osseointegrated prostheses for patients with unilateral transfemoral amputation at two-year follow-up. Arch Phys Med Rehabil 2014; 95: 2120–2127. DOI: 10.1016/j.apmr.2014.07.009 25064778

[40] Leon AC, Davis LL, Kraemer HC. The role and interpretation of pilot studies in clinical research. J Psychiatr Res 2011; 45: 626–629. DOI: 10.1016/j.jpsychires.2010.10.008 21035130 PMC3081994

[41] Bell JC, Wolf EJ, Schnall BL, Tis JE, Tis LL, Potter BK. Transfemoral amputations: the effect of residual limb length and orientation on gait analysis outcome measures. J Bone Joint Surg Am 2013; 95: 408–414. DOI: 10.2106/JBJS.K.01446 23467863

[42] Lu CF, Liu YC, Yang YR, Wu YT, Wang RY. Maintaining gait performance by cortical activation during dual-task interference: a functional near-infrared spectroscopy study. PLoS One 2015; 10: e0129390. DOI: 10.1371/journal.pone.0129390 26079605 PMC4469417

[43] Lin MI, Lin KH. Walking while performing working memory tasks changes the prefrontal cortex hemodynamic activations and gait kinematics. Front Behav Neurosci 2016; 10: 92. DOI: 10.3389/fnbeh.2016.00092 27242461 PMC4870471

[44] Cooper RJ, Selb J, Gagnon L, Phillip D, Schytz HW, Iversen HK, et al. A systematic comparison of motion artifact correction techniques for functional near-infrared spectroscopy. Front Neurosci 2012; 6: 147. DOI: 10.3389/fnins.2012.00147 23087603 PMC3468891

[45] Tak S, Ye JC. Statistical analysis of fNIRS data: a comprehensive review. Neuroimage 2014; 85 Pt 1: 72–91. DOI: 10.1016/j.neuroimage.2013.06.016 23774396

