# Supplementary material for: COGNITIVE LOAD IN INDIVIDUALS WITH A TRANSFEMORAL AMPUTATION DURING SINGLE- AND DUAL-TASK WALKING: A PILOT STUDY OF BRAIN ACTIVITY IN PEOPLE USING A SOCKET PROSTHESIS OR A BONE-ANCHORED PROSTHESIS
Source: J Rehabil Med. 2024 Aug 22;56:40111. doi: 10.2340/jrm.v56.40111 (PMC11358844; doi:10.2340/jrm.v56.40111)

Supplementary material has been published as submitted. It has not been copyedited, or typeset by Journal of Rehabilitation Medicine

Fig. S1. Cortical brain activity (millimole of oxygenated haemoglobin, HbO_2) in the left and right regions of interest during level walking for 10 seconds. Y axis represents the relative concentration of HbO_2 (mM). X axis represents time (sec). S=socket prosthesis users BA= Bone anchored prosthesis users.

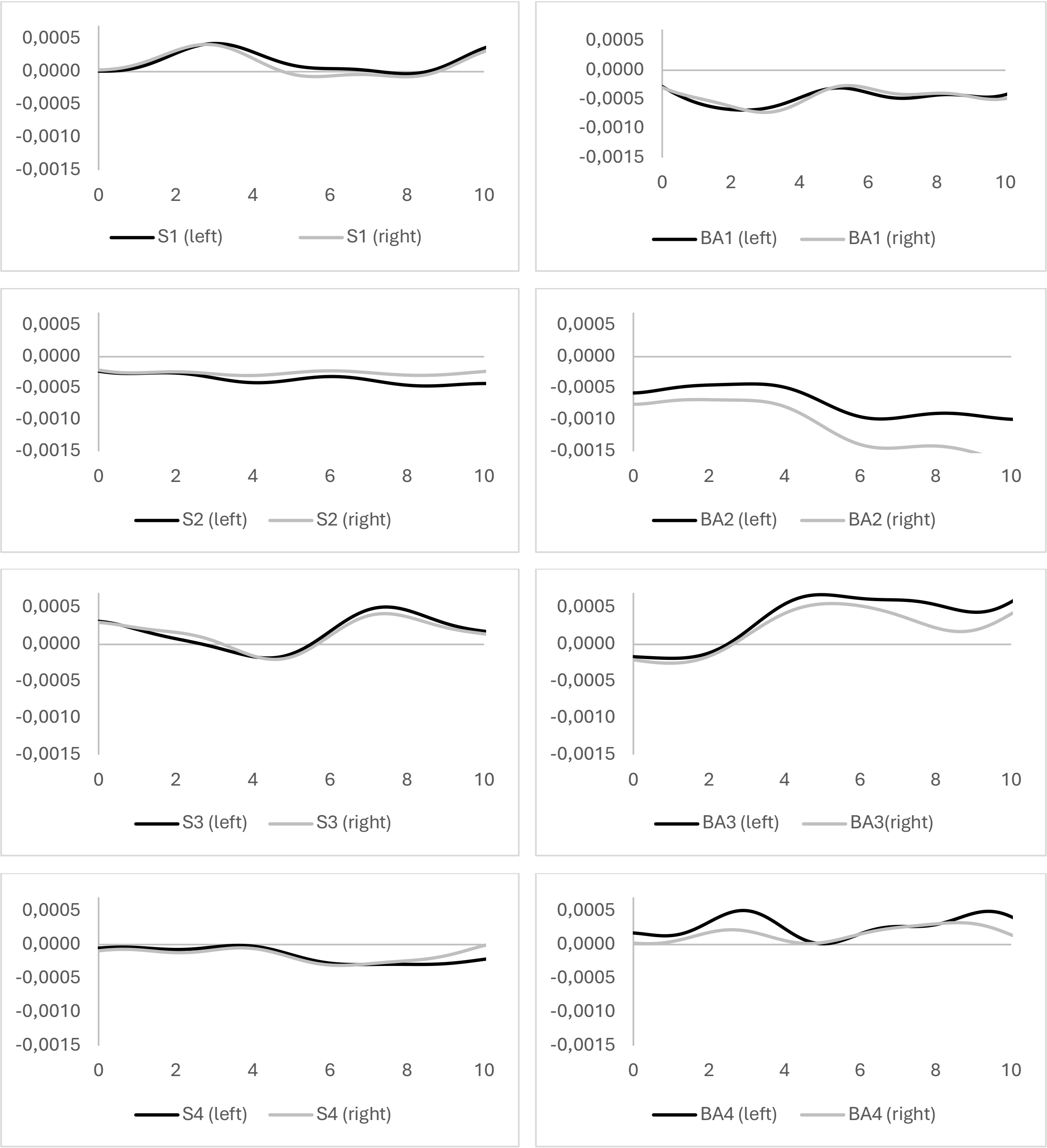

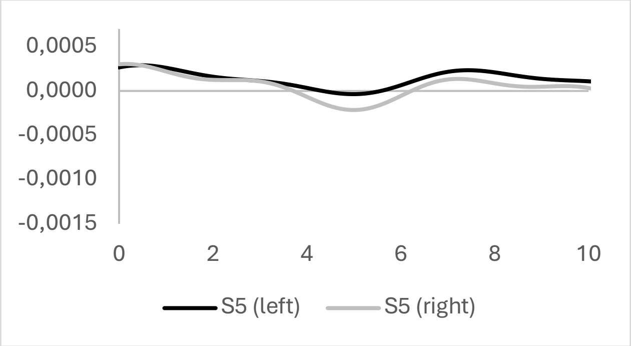
0,0005
0,0000
-0,0005
-0,0010
-0,0015
0
2
4
6
8
10
S5 (left)
S5 (right)

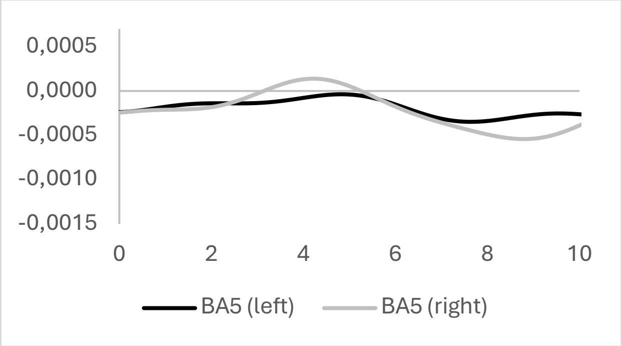
0,0005
0,0000
-0,0005
-0,0010
-0,0015
0
2
4
6
8
10
BA5 (left)
BA5 (right)

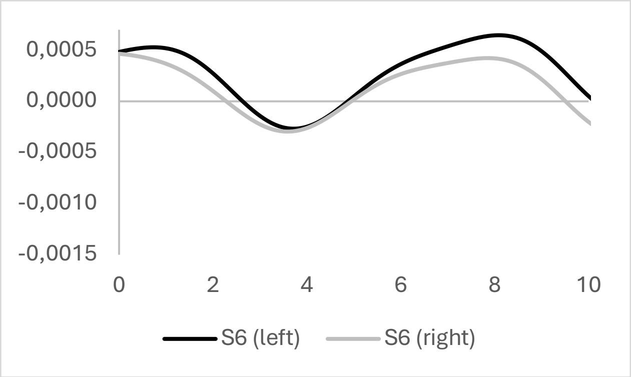
0,0005
0,0000
-0,0005
-0,0010
-0,0015
0
2
4
6
8
10
S6 (left)
S6 (right)

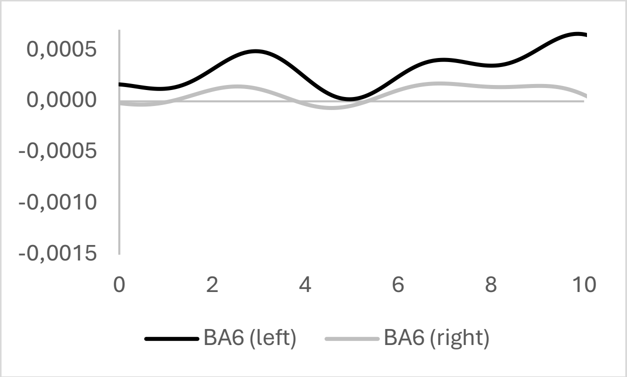
0,0005
0,0000
-0,0005
-0,0010
-0,0015
0
2
4
6
8
10
BA6 (left)
BA6 (right)

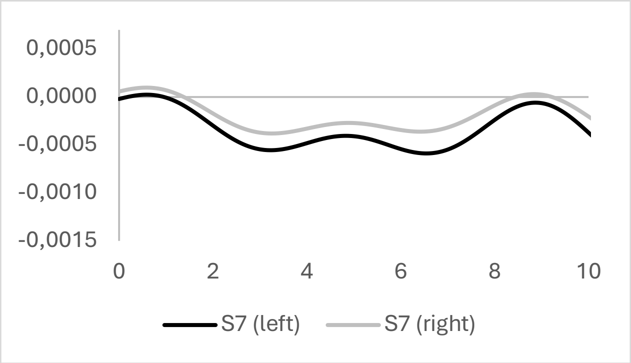
0,0005
0,0000
-0,0005
-0,0010
-0,0015
0
2
4
6
8
10
S7 (left)
S7 (right)

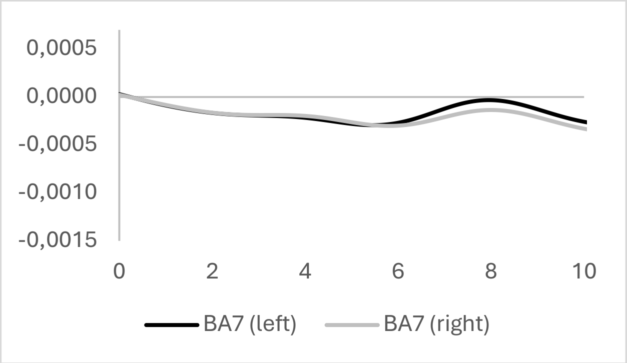
0,0005
0,0000
-0,0005
-0,0010
-0,0015
0
2
4
6
8
10
BA7 (left)
BA7 (right)

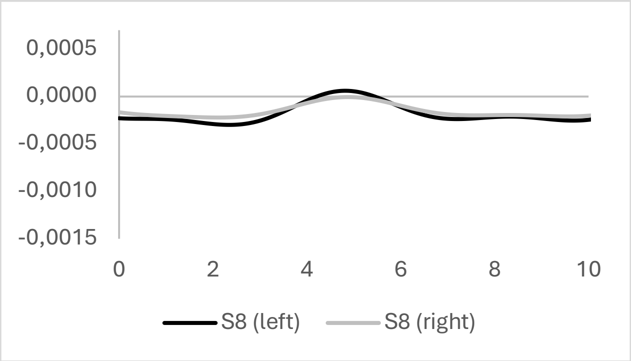
0,0005
0,0000
-0,0005
-0,0010
-0,0015
0
2
4
6
8
10
S8 (left)
S8 (right)

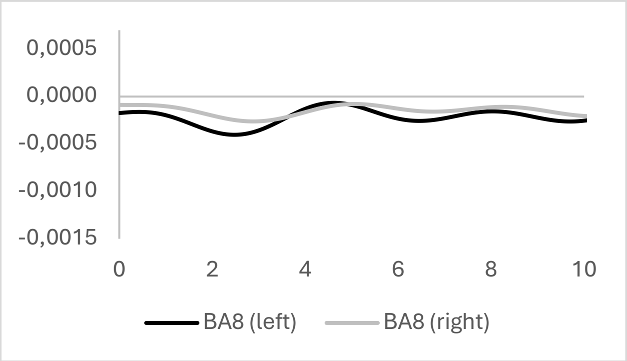
0,0005
0,0000
-0,0005
-0,0010
-0,0015
0
2
4
6
8
10
BA8 (left)
BA8 (right)

Fig. S2. Cortical brain activity (millimole of oxygenated haemoglobin, HbO_2) in the left and right regions of interest during the key-test for 10 seconds. Y axis represents the relative concentration of HbO_2 (mM). X axis represents time (sec). S=socket prosthesis users BA= Bone anchored prosthesis users. Note S8 missing data.

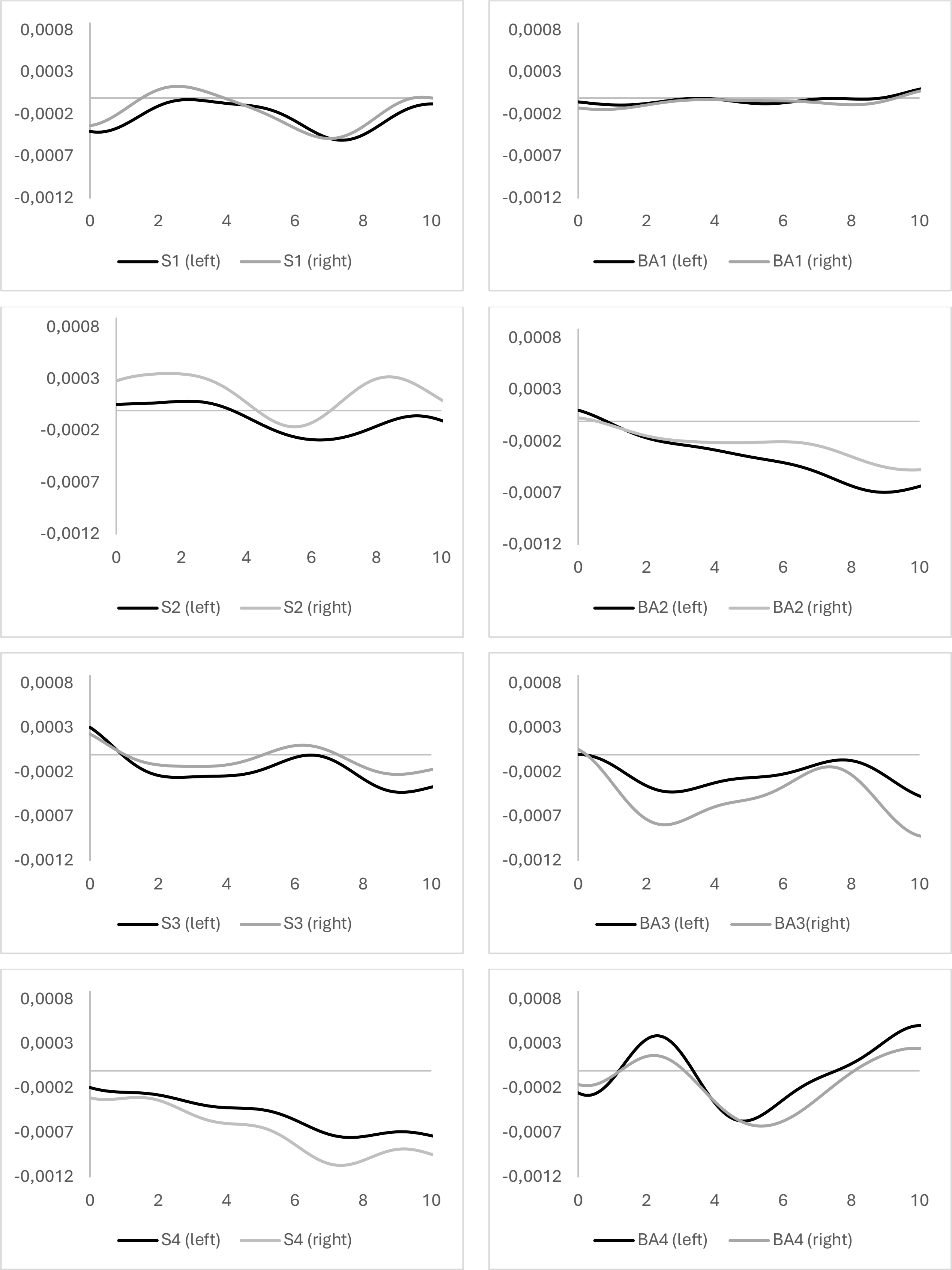

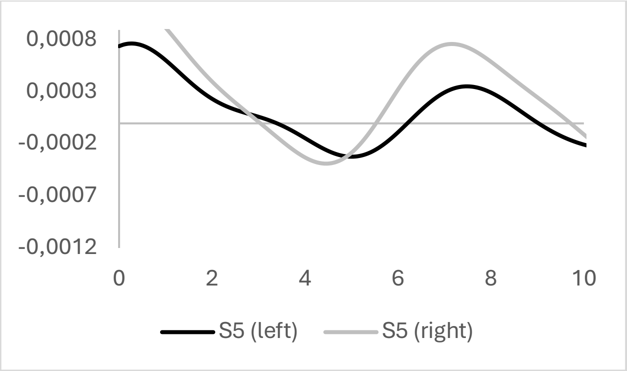
0,0008
0,0003
-0,0002
-0,0007
-0,0012
0
2
4
6
8
10
S5 (left)
S5 (right)

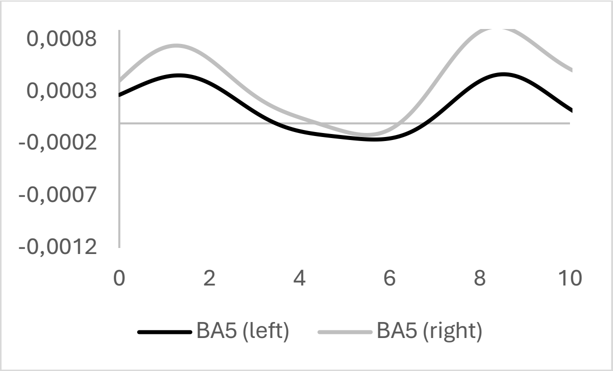
0,0008
0,0003
-0,0002
-0,0007
-0,0012
0
2
4
6
8
10
BA5 (left)
BA5 (right)

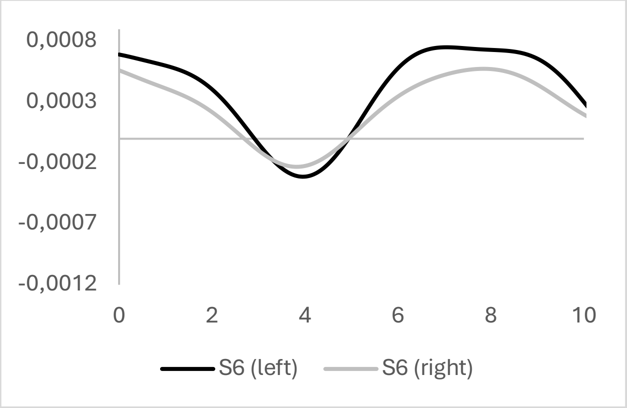
0,0008
0,0003
-0,0002
-0,0007
-0,0012
0
2
4
6
8
10
S6 (left)
S6 (right)

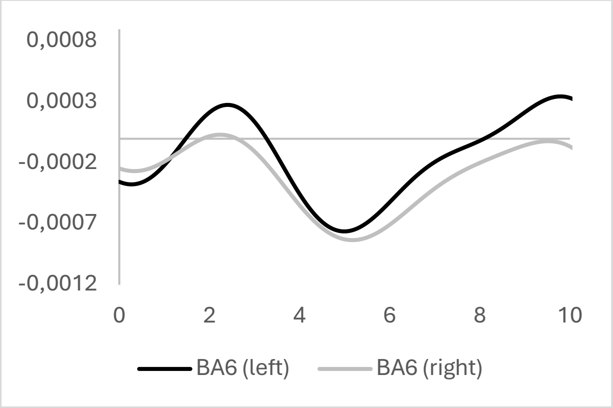
0,0008
0,0003
-0,0002
-0,0007
-0,0012
0
2
4
6
8
10
BA6 (left)
BA6 (right)

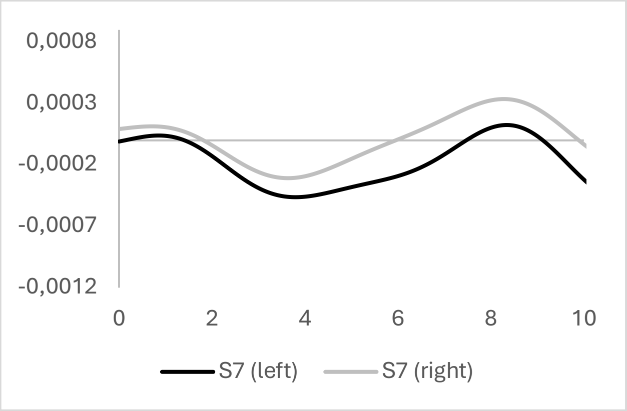
0,0008
0,0003
-0,0002
-0,0007
-0,0012
0
2
4
6
8
10
S7 (left)
S7 (right)

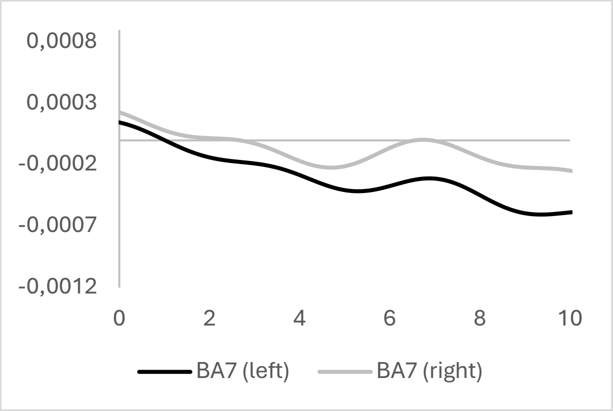
0,0008
0,0003
-0,0002
-0,0007
-0,0012
0
2
4
6
8
10
BA7 (left)
BA7 (right)

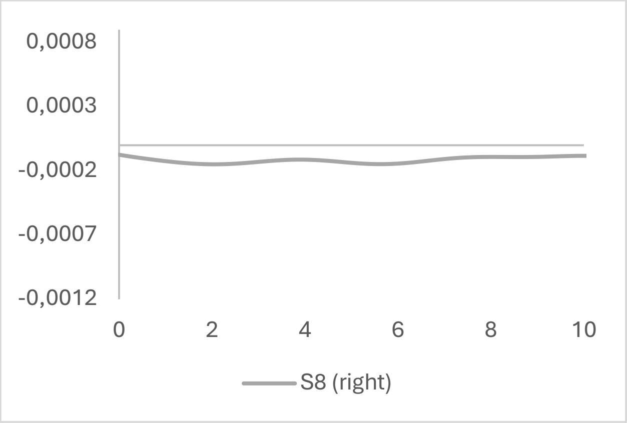
0,0008
0,0003
-0,0002
-0,0007
-0,0012
0
2
4
6
8
10
S8 (right)

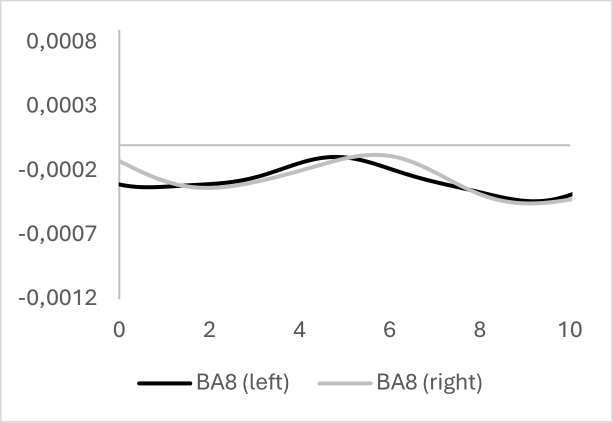
0,0008
0,0003
-0,0002
-0,0007
-0,0012
0
2
4
6
8
10
BA8 (left)
BA8 (right)

Fig. S3. Cortical brain activity (millimole of oxygenated haemoglobin, HbO_2) in the left and right regions of interest during the trail-walking test, TWT for 10 seconds. Y axis represents the relative concentration of HbO_2 (mM). X axis represents time (sec). S=socket prosthesis users BA= Bone anchored prosthesis users. Note S8 and BA8 missing data.

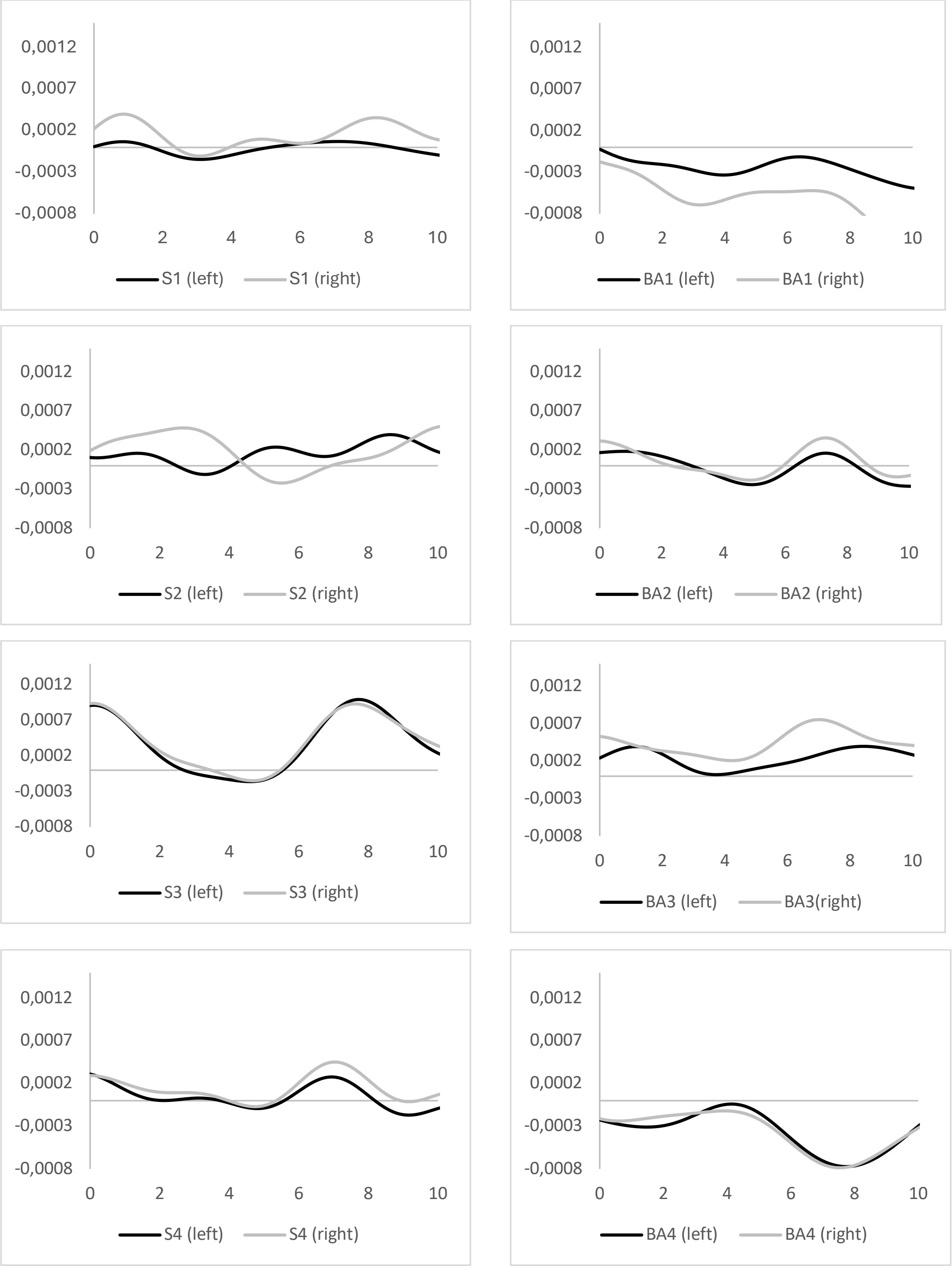

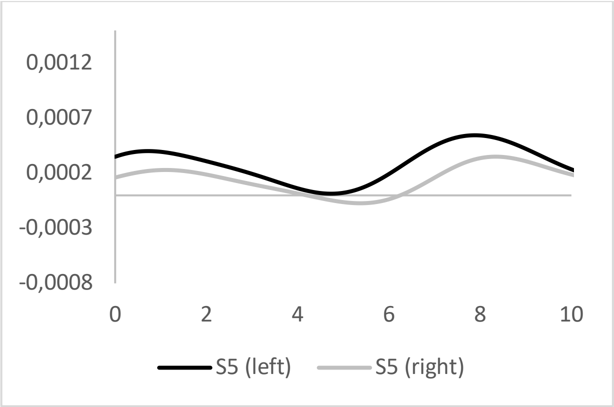

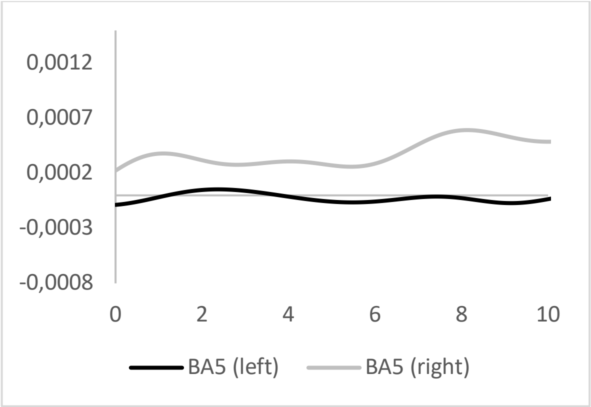

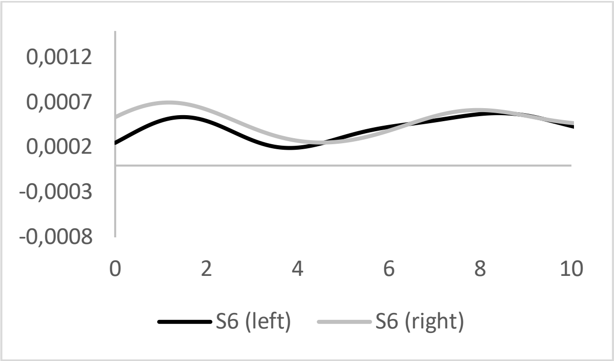

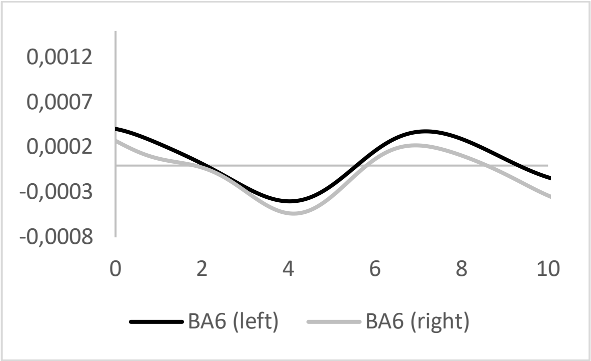

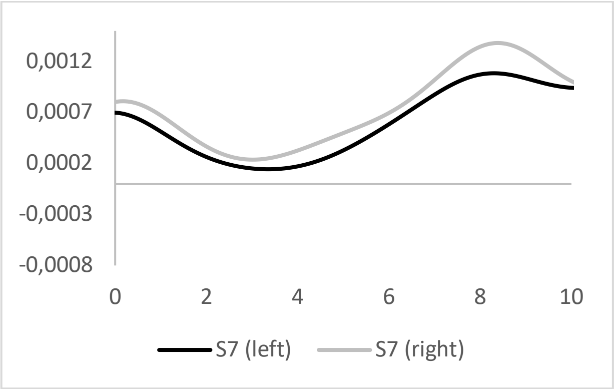

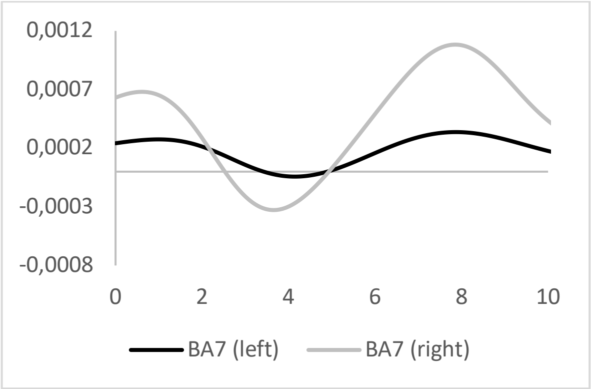

Missing data

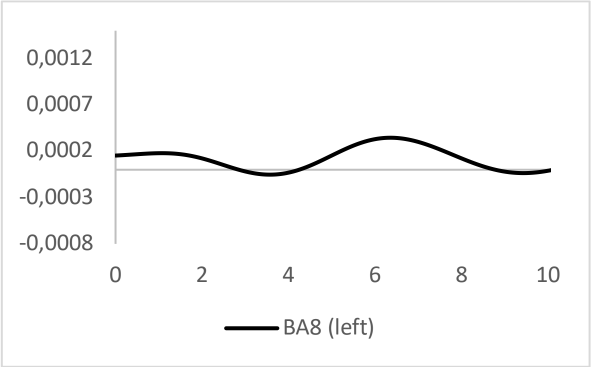

Supplement: COGNITIVE LOAD IN INDIVIDUALS WITH A TRANSFEMORAL AMPUTATION DURING SINGLE- AND DUAL-TASK WALKING: A PILOT STUDY OF BRAIN ACTIVITY IN PEOPLE USING A SOCKET PROSTHESIS OR A BONE-ANCHORED PROSTHESIS [file JRM-56-40111-s1.pdf]
